# Advances in machine learning applications for cardiovascular 4D flow MRI

**DOI:** 10.3389/fcvm.2022.1052068

**Published:** 2022-12-09

**Authors:** Eva S. Peper, Pim van Ooij, Bernd Jung, Adrian Huber, Christoph Gräni, Jessica A. M. Bastiaansen

**Affiliations:** ^1^Department of Diagnostic, Interventional and Pediatric Radiology (DIPR), Inselspital, Bern University Hospital, University of Bern, Bern, Switzerland; ^2^Translational Imaging Center (TIC), Swiss Institute for Translational and Entrepreneurial Medicine, Bern, Switzerland; ^3^Department of Radiology and Nuclear Medicine, Amsterdam University Medical Centers, Amsterdam, Netherlands; ^4^Department of Pediatric Cardiology, Wilhelmina Children’s Hospital, University Medical Center Utrecht, Utrecht, Netherlands; ^5^Department of Cardiology, Inselspital, Bern University Hospital, University of Bern, Bern, Switzerland

**Keywords:** 4D flow cardiovascular magnetic resonance, 4D flow, four-dimensional flow imaging, artificial intelligence, machine learning (ML)

## Abstract

Four-dimensional flow magnetic resonance imaging (MRI) has evolved as a non-invasive imaging technique to visualize and quantify blood flow in the heart and vessels. Hemodynamic parameters derived from 4D flow MRI, such as net flow and peak velocities, but also kinetic energy, turbulent kinetic energy, viscous energy loss, and wall shear stress have shown to be of diagnostic relevance for cardiovascular diseases. 4D flow MRI, however, has several limitations. Its long acquisition times and its limited spatio-temporal resolutions lead to inaccuracies in velocity measurements in small and low-flow vessels and near the vessel wall. Additionally, 4D flow MRI requires long post-processing times, since inaccuracies due to the measurement process need to be corrected for and parameter quantification requires 2D and 3D contour drawing. Several machine learning (ML) techniques have been proposed to overcome these limitations. Existing scan acceleration methods have been extended using ML for image reconstruction and ML based super-resolution methods have been used to assimilate high-resolution computational fluid dynamic simulations and 4D flow MRI, which leads to more realistic velocity results. ML efforts have also focused on the automation of other post-processing steps, by learning phase corrections and anti-aliasing. To automate contour drawing and 3D segmentation, networks such as the U-Net have been widely applied. This review summarizes the latest ML advances in 4D flow MRI with a focus on technical aspects and applications. It is divided into the current status of fast and accurate 4D flow MRI data generation, ML based post-processing tools for phase correction and vessel delineation and the statistical evaluation of blood flow.

## Introduction

Since its emergence in 1993 (1–3), four-dimensional (4D) flow magnetic resonance imaging (MRI) has evolved as a non-invasive imaging technique to visualize and quantify blood flow and has been used for clinical imaging since the early 2000s (4, 5). 4D flow MRI is based on a time-resolved 3D phase contrast MRI sequence and is widely applied to the heart and vessels.

The quantification of net flow and peak velocities from 4D flow MRI has shown to be of diagnostic relevance for cardiovascular diseases such as the grading of stenoses, aortic coarctation, or aortic- and mitral valve regurgitation (6–9). Also, the visualization of the direction of the blood flow is important, for example in aortic aneurysms, aortic dissections and coarctations, in hypertrophy cardiomyopathy (6, 10), as well as in congenital heart disease, such as univentricular hearts or transposition of the great arteries (11, 12). Moreover, 4D flow MRI allows the direct quantification of regurgitant flow compared to traditional indirect methods (i.e., subtracting stroke volume calculated from aortic 2D flow MRI from stroke volume measured by left ventricular segmentation) in mitral valve insufficiency (13). Furthermore, 3D visualization of the blood flow using pathlines can help interpreting complex flow patterns pre- and post-surgery, such as the Fontan procedure (14). Also, other biomarkers such as kinetic energy (KE) (15, 16), turbulent kinetic energy (TKE) (17, 18), viscous energy (VE) loss (16), wall shear stress (WSS) (19, 20) or pulse wave velocity (PWV) (21) have shown significant differences in patients with cardiovascular disease compared to normal subjects.

Four-dimensional flow MRI, however, has several limitations. Due to its velocity encoding scheme, 4D flow MRI takes at least four times as long as cine MRI scans (i.e., around 10 min). This poses limits on the clinical application due to additional costs, patient discomfort and motion artifacts. Additionally, limited spatio-temporal resolutions, constrained by the signal-to-noise-ratio (SNR) and scan time, lead to inaccuracies in velocity measurements in small vessels, low-flow venous vessels and near the vessel wall due to partial volume effects (14). This in turn creates inaccurate grading of stenoses and inaccuracies in WSS estimation (22, 23). Additionally, 4D flow MRI is subject to inherent inaccuracies of the MRI measurement process such as residual phase errors, induced by eddy currents, concomitant fields, or even mechanical vibrations (24), which can lead to errors in velocity estimations. Although tuning of the scanners’ pre-emphasis can help to correct for non-linearities in the gradient field, these inaccuracies, as well as phase aliasing effects, must be corrected for retrospectively, creating long post-processing times using dedicated software. The post-processing times are prolonged as net flow and peak velocities are typically evaluated by (manually) placed 2D planes and contours at the location of the corresponding vessel or valve within the 3D acquisition. Parameters such as KE, VE, TKE and WSS require even a careful delineation of the 3D vessel lumen.

Various machine learning (ML) techniques have been proposed to overcome these limitations. Existing scan acceleration methods, such as compressed sensing (CS) (25–27) have been extended using ML reconstructions which are able to speed up the image reconstruction time up to a couple of seconds (28). Also, ML super-resolution methods can assimilate high-resolution computational fluid dynamic (CFD) simulations and 4D flow MRI, which leads to more realistic velocity results. ML based techniques, such as U-Nets, used to localize vessels and segment vessel boundaries, have been applied to 4D flow MRI to automate contour drawing and 3D segmentation. ML efforts have also focused on the automation and acceleration of other post-processing steps, by learning phase corrections and anti-aliasing.

This review summarizes the latest ML advances in 4D flow MRI with a focus on technical aspects and applications, including all original research articles published on the topics of (4D) flow MRI and ML published until November 2022. It is divided into the current status of (1) scan acceleration and image reconstruction, (2) super resolution and data assimilation for fast and accurate 4D flow MRI data generation, as well as ML based post-processing methods for (3) phase corrections, (4) vessel segmentation and (5) the statistical evaluation of blood flow.

## Scan acceleration and image reconstruction

**FIGURE 1:**
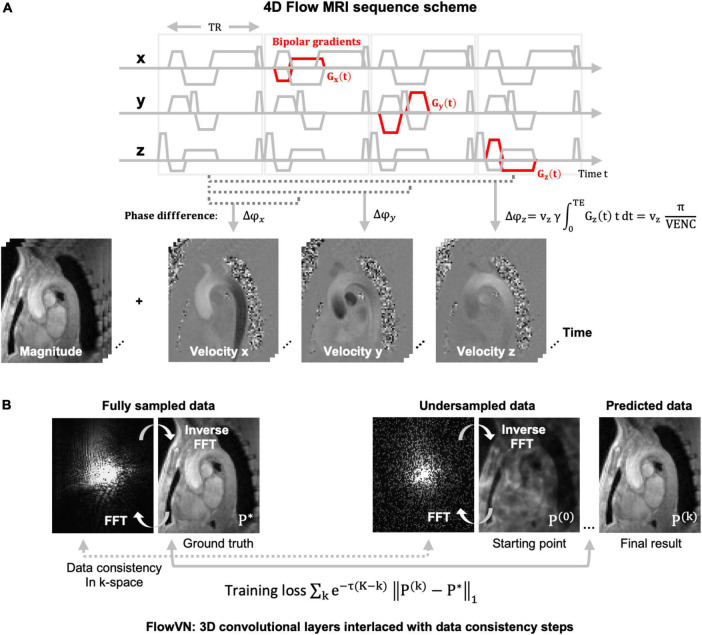
**(A)** 4D flow MRI image acquisition scheme using a spoiled gradient echo sequence with additional bipolar gradients for motion encoding (red). The velocity maps of this exemplary 4D flow MRI dataset of the aortic arch are obtained by subtracting the phase ϕ of the reference and the three flow encoded images. Magnitude images for the four acquired images are averaged, or in other applications, used for turbulence encoding. All images are time-resolved, representing one cardiac cycle. The velocity encoding strength (VENC) in [cm/s] is set by the user and is inversely proportional to the area of the bipolar gradient. The VENC is usually chosen in the range of the maximum expected velocity to prevent velocity aliasing. **(B)** Fully sampled and randomly undersampled k-space and their corresponding images. The undersampled data displays incoherent artefacts in image space (P^(0)^). In data recovery training with the FlowVN (28). P^(0)^ is the Fourier transform of the undersampled k-space and the starting point of the training. The training loss is defined by the difference of the reconstructed image with the fully sampled data (P*). The data consistency is calculated in k-space as the difference between the sampled datapoints. After k iterations image P^(k)^ is achieved. Abbreviations: TR = repetition time, TE = echo time, x,y,z = spatial dimension, v = velocity, γ = gyromagnetic ratio, FFT = fast Fourier transform, P = image, k = iteration steps.

4D flow MRI uses additional magnetic field gradients to encode the velocity of moving blood. These gradients are applied to each spatial direction separately, which results in four different images, the reference image and three flow encoded images, also called 4-point encoding (Figure 1A). As the scan time is therefore four times as long, various acceleration techniques have been proposed (25, 29–32). These acceleration methods skip datapoints in k-space (undersampling), which creates aliasing artifacts in the image when using a conventional reconstruction. Most image reconstruction algorithms of these techniques take advantage of information redundancies – similar to those used for image compression – such that the full information content can be derived (27). However, the runtimes for those (iterative) reconstruction algorithms range between 10 and 60 mins, a drawback that can be tackled with machine learning (ML) approaches.

Most approaches for ML image reconstruction are based on artifact-removal of undersampled data in image space, rather than training a network to retrieve the full image content directly from the undersampled k-space. In 2019, Vishnevsky et al. (28) implemented a variational neural network (FlowVN) for fast, automatic image reconstruction of undersampled 4D flow MRI data. During training, fully sampled data served as a ground truth and was retrospectively undersampled using a random undersampling pattern as used in CS (33–37) applications (see Figure 1B). The starting point of the FlowVN training was an image with random, noise-like undersampling artifacts (the Fourier transform of a randomly undersampled k-space) as shown in Figure 1B. The network used this image, the undersampled k-space data (real and imaginary parts), and the coil sensitivity maps as inputs for training. It consisted of 3D convolutional layers and data consistency steps. This design [similar to Hammerik et al. (38)] enabled that (1) the network learns differences between the ground truth and the artifact image and (2) that the data points in k-space for sampled and undersampled data match. The output were artifact-free images close to the fully sampled data. The network could demonstrate its similar performance to a regular CS image reconstruction; however, the runtime was 21 s for the FlowVN vs. 10 min for the CS reconstruction. Also, when applied to 13 times undersampled patient data, the FlowVN was 30 times faster and systolic peak velocity errors were only marginally lower (–1.59% for FlowVN and –1.18% for CS). In a different study, Haji-Valiyadeh et al. (39) used a 3D U-Net to remove aliasing artifacts from undersampled radial 2D flow MRI data for the purpose of fast, real-time data acquisition. They developed a network trained on 510 radial, real-time 2D flow datasets, which were artificially created from the images of Cartesian 2D flow dataset. The undersampling artifact removal of the network was then tested in an actual free-breathing real-time 2D flow sequence for acceleration factors up to 28. In a comparison to a CS reconstruction of the real-time data the 3D U-net filtering was almost 5 times faster and could recover higher peak velocity values than the CS reconstruction. Peak velocity values were also closer to the ground truth of non-real-time image acquisition, represented by an average heartbeat composed of by all the heartbeats throughout the acquisition, when compared with the CS reconstruction. Another way of 4D flow MRI scan acceleration was recently suggested by Kim et al. (40). The proposed network learns to recover velocity maps as obtained by regular 4-point encoding (referring to 4 acquisitions, as illustrated in Figure 1A) by replacing it with a sampling scheme that requires only three acquisitions and learning the phase reconstruction subsequently. Velocity results demonstrated a good agreement between both encoding schemes (regression slope = 0.96 and *R*^2^ = 0.992).

## Super resolution and data assimilation

To increase the spatio-temporal resolution of 4D flow MRI, which is limited by SNR and scan time, ML super-resolution techniques can be applied. These techniques learn on paired high- and low-resolution datasets to resolve an image resolution higher than the input resolution. As there is typically a lack of high-resolution *in vivo* data, most super-resolution approaches for 4D flow MRI rely on synthetic images created by CFD simulations. These simulations solve the Navier Stokes equation in a given vessel geometry and under given inflow conditions and can be computed at resolutions much higher than the maximum achievable resolutions with 4D flow MRI, while maintaining correct physics.

**FIGURE 2:**
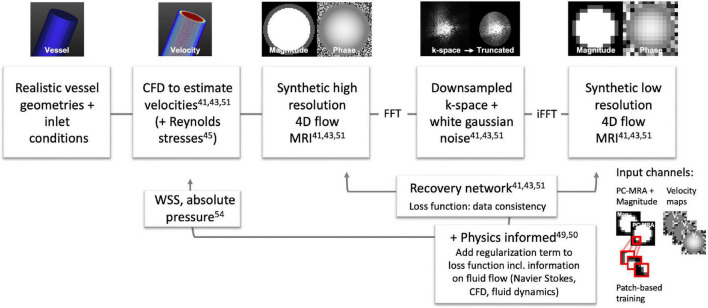
Overview of frameworks for 4D flow MRI super-resolution training and data assimilation for 4D flow MRI and CFD data: Ferdian et al. (aortic simulation) (41), Rutkowski et al. (cerebrovascular simulations) (43), Kissas et al. (49), Fathi et al. (50), Dirix et al. (45), Ferdian et al. (54). Exemplary data in this figure was created with GTFlow (Gyrotools, Zürich, Switzerland).

In 2020, Ferdian et al. (41) developed a framework to derive synthetic high-resolution 4D flow MRI images from CFD simulations in the aorta for training a super-resolution network. They used three aortic geometries to generate simulations with high spatial resolution and a temporal resolution of 71 cardiac frames, using inlet and outlet conditions at the ascending and descending aorta. From the simulations synthetic 4D flow MRI images were generated by deriving the velocity fields and dividing them into their spatial v_x_, v_y_, and v_z_ components, similar to a 4D flow MRI acquisition (Figure 2). Then, a complex signal was created with the velocity maps as the signals phase and a simulated magnitude, followed by a fast Fourier transform (FFT) to generate a synthetic k-space (Figure 2). To mimic MRI characteristics the CFD data was down-sampled in k-space i.e., high frequency components were cut off, and Gaussian noise was added to the complex signal to achieve a pre-defined SNR. After inverse fast Fourier transform (IFFT) the image represented a complex, MRI-like low-resolution signal. A super-resolution residual network (4DFlowNet), based on the generator of the SRResNet network (42), was then trained to recover the high-resolution data. Training was performed on the paired synthetic high- and low-resolution 4D flow MRI data. The input layers consisted of two parts, the anatomical one (with channels: phase-contrast magnetic resonance angiogram and magnitude image), and the velocity one (with channels: v_x_, v_y_, and v_z_ velocity maps). In this setup, the anatomical channels selected the vessel regions and supported de-noising. As only three aortic geometries were available, the network training was patch-based, that means 16 × 16 × 16 randomly selected voxel patches, with a flow region of at least 20%, were used for training. The super-resolution network could successfully recover simulated data with a resolution down-sampled by a factor of 2 and at varying SNR levels. The network was then applied to high-resolution phantom and high- (2 mm) and low-resolution (4 mm) volunteer 4D flow MRI datasets. The study showed that the super-resolution network had smaller flow rate errors averaged in an ROI at in- (–0.6%) and outlet (5.8%) than interpolated data at in- (7%) and outlet (5.8%) in the phantom and (1.1%) and (3.8%) *in vivo*. In a similar study, Rutkowski et al. (43) used high-resolution, CFD-derived vector fields to create synthetic, MRI like, high- and low-resolution data pairs. CFD simulations were calculated on cerebrovascular flow models of five patient-specific aneurysms on which data augmentation (changes in diameter size, aneurysm geometry, synthetic vessel creation) was applied. The vessels had rigid walls and a time-resolved inflow profile as an inlet condition. Simulations were repeated 6 times with different inflow profiles, which led to 180 unique time-varying velocity fields. For training, a CNN similar to standard super-resolution networks (44) was used. 32 × 32 × 32 velocity field blocks were extracted from the simulated MRI acquisition. The loss function was based on magnitude weighted least squares and the network was tested in retrospectively down-sampled phantom data, allowing for a comparison against the original high-resolution dataset. Also, the network was applied to 20 time-averaged 4D flow MRI patient datasets (0.4–0.6 mm isotropic spatial resolution, 20 frames). As a result, the network could remove background noise up to 64%. Overall, the 4D flow MRI derived velocities had lower noise and a higher spatial resolution when enhanced with the CNN. Vessel boundaries could be delineated better, and the velocities close to the walls were estimated more accurately, including smoother velocity gradients near the wall. In the future, these simulations and ML frameworks might be extended to more advanced 4D flow MRI acquisition schemes, including turbulence induced signal dephasing in the magnitude images. Dirix et al. (45) developed a similar framework for synthesized 4D flow MRI images using multipoint encoding to achieve turbulence assessment (45).

For ML it can be advantageous (faster, more accurate, less training data) to restrict the space of solutions. Generally, data fidelity terms in the loss function of neural networks minimize the distance between the predicted output and the measured data. Physics-informed networks include a regularization part that enforces the underlying physical principles of a given dataset. For 4D flow MRI this can for example be the conservation of mass and momentum in the flow domain, which leads to a correct solution even with limited training data (46). In contrast, other non-machine learning-, but physics-based methods use divergence free velocity fields as a constraint to 4D flow MRI data (47) and CFD based velocity field optimizations to inform the 4D flow MRI data about CFD physics (48). The physics informed neural network introduced by Raissi et al. (46) was picked up by Kissas et al. (49) and Fathi et al. (50) in 2020 for 4D flow MRI implementations. The network from Kissas et al. (49) solves partial differential equations using a neural network to predict flow and pressure from 4D flow MRI measurements of the carotid bifurcation. It was trained on simulations with 1D Navier Stokes equations. Fathi et al. (50) trained a deep neural network with the aim to remove noise and to increase the resolution of 4D flow MRI data. They restricted the space of solutions of the applied network by a regularization term on the Navier Stokes equations within a pre-defined region inside the blood flow. The data fidelity term (the same as in (46)) was applied to the entire data. The network then output v_x_, v_y_, and v_z_ velocity components, pressure, and the magnitude image. The network was trained using synthetic 4D flow MRI data and tested on 4D flow MRI scans of a silicon phantom. For their workflow only a rough segmentation of the blood flow region was necessary (in which Navier Stokes was valid), and in contrast to other techniques, no strict boundaries or inflow conditions had to be defined, which made it less error prone. They could demonstrate a significant reduction in velocity errors during simulation, however, phantom measurements showed marginal improvements of velocity estimation. Very recently, a super resolution 4D flow network (SRflow) has been published by Shit et al. (51) in which they achieved a higher velocity-to-noise ratio in images with a 4-times increased resolution using their super-resolution approach than using a regular cubic B-spline interpolation.

4D flow derived biomarkers, such as WSS, have been associated with endothelial cell remodeling, for regions of low WSS (or high oscillatory WSS) in particular. Also, high WSS has been associated with disease patterns such as in aortic stenosis and aortic dissection. However, limited spatial resolution, partial volume effects and segmentation inaccuracy do not allow for accurate WSS, which is typically solved with curve-fitting and interpolation (52, 53). 4D flow MRI derived WSS therefore typically results in an underestimation when compared to CFD (22). Ferdian et al. (54) developed a U-Net based ML network (WSSNet) to directly estimate WSS from 4D flow MRI, trained on patient-specific CFD simulations and synthetic 4D flow MRI. The datasets consisted of 37 aortic geometries and simulated velocities. The input of the WSSNet were 2D maps of simulated velocities close to the vessel border and their coordinates with respect to the border. The network learned the connection between geometry, velocity and WSS, and the output were estimated WSS values (which were compared to WSS values calculated from the CFD data). To generalize better to 4D flow MRI, synthetic low-resolution 4D flow MRI was created from the CFD data and the training repeated. Then the network was applied to 43 real, *in vivo* 4D flow MRI datasets and compared against the fitting algorithms for WSS estimation. The mean absolute error of the estimated WSS using the network was 0.55 ± 0.60 Pa (relative error 4.34 ± 4.14%). The values correlated well with the WSS from the CFD simulations, reporting a correlation coefficient of *r* = 0.92 ± 0.05. The estimated WSS showed 2–3 times higher WSS values when compared to regular fitting methods and more robustness to artificially introduced noise.

## Phase corrections

Since velocity maps are derived from the phase of the 4D flow MRI signal, sources that introduce phase offsets, such as eddy currents, can impair the data quality. Phase corrections and anti-aliasing can be performed retrospectively to the acquisition but are user-dependent and time-consuming. ML techniques, however, can learn and apply these corrections.

Eddy current induced background phase can be corrected for by linear or polynomial fits of the phase in static tissue regions. The calculated phase error fields can then be applied the flow regions to correct the estimated velocities. You et al. (55) used 139 (85 training, 14 validation, 40 testing) abdominopelvic 4D flow MRI datasets to train a multichannel 3D U-Net that automatically generates phase error fields for correction. Flow analysis was performed on the testing datasets and compared against a regular background phase correction as a reference, which included a manual detection of static tissue regions using dedicated software. Assuming in- and outflow values to be the same, non-corrected images showed an offset due to background fields and a low correlation between in- and outflow values. The Pearson’s correlation coefficient *r* was reported to be *r* = 0.5, with a *p*-value of *p* < 0.001. After manual correction this increased to *r* = 0.98, *p* < 0.001 and after automatic ML correction to *r* = 0.91, *p* < 0.001. Flow differences reduced from uncorrected –0.14 L/min to corrected 0.05 L/min for regular and ML correction. This technique demonstrated the use of a fast, automated correction and the feasibility of ML training for this task, demonstrating similar results as manual correction. However, also (semi-) automatic algorithms for the selection of static tissue regions and fitting exists (and usually perform well), which were not included as a reference in the study.

**FIGURE 3:**
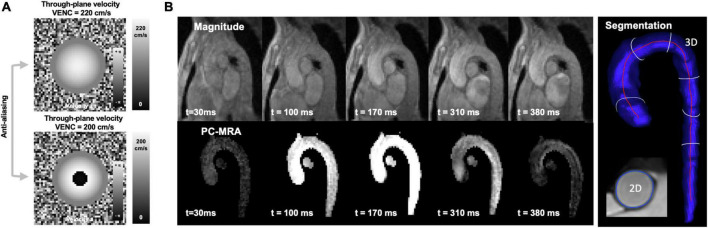
**(A)** Simulated parabolic flow with a central peak velocity of 220 cm/s and the aliasing effect create in the phase-difference image and the velocity map when a lower VENC e.g., of 200 cm/s is chosen. **(B)** Temporal evolution of the magnitude and PC-MRA signal in an aortic 4D flow MRI dataset. Magnitude images do not allow for fast segmentations based on thresholding as blood and tissue have a similar contrast. PC-MRA images lose their signal when there is no apparent blood flow e.g., during diastole.

Aliasing effects, or phase-wraps, can occur if the velocity encoding, defined by the VENC, was chosen too low. High velocities, higher than the VENC value, will appear as wrapped phases (transitioning from + π to –π) in the velocity map (Figure 3A), which must be corrected for retrospectively. The correction, however, requires the identification of the aliased voxel in 3D and for all time frames. There are several semi-automatic solutions that support 2D voxel wise un-wrapping by region-merging and graph cut optimization (56, 57), which, however, require a start point for unwrapping or rely on spatio-temporal smoothness (58, 59). These methods were also adapted to be applicable to 4D flow MRI by using a Laplacian algorithm (60). Robust, automatic detection of all aliased voxel in all time frames, however, remains challenging and large, aliased regions or regions with multiple wraps remain a problem. In Berhane et al. (61) a U-Net CNN was used to automatically detect and correct aliasing in 667 4D flow dataset (VENCs ranging between 60 and 500 cm/s, 534 with contrast agent, 321 bicuspid aortic valve (BAV), 247 tricuspid aortic valve, 99 controls). Aliasing was either introduced during acquisition or retrospectively added. An additional 10 subjects were acquired with three different VENC settings (60, 100, 175 cm/s) to show the accuracy of the unwrapping method. From all datasets static segmentations of the thoracic aorta were created. Datasets without aliasing (*N* = 305) were used to introduce aliasing in predefined regions, serving as labeled pairs of ground truth and aliased voxels. The data was split up in training (and validation) and testing, with a binary mask for the aliased voxels as a network output. Test results provided much better correction when compared to an automated method (from Salfity (58, 59)). The difference of the performance of the techniques was significant with a Dice score (DS) between 0.89 and 0.99 (for the different VENCs) for the CNN and between 0.84 and 0.90 for the conventional algorithm. Ten datasets scanned with different VENCs showed similar peak velocity, net and peak flow rates for the conventional anti-aliasing algorithm and the CNN corrected datasets. However, no comparison against a 4D Laplacian algorithm was done and also multiple phase wrapping was not taken into account. Also, phase-unwrapping at the vessel wall was limited, which leaves the phase-unwrapping problem open to find a fully automatic solution.

## Vessel segmentation

4D flow MRI requires accurate delineation of the vessel lumen for calculation of mean velocities, flow and WSS. The blood-tissue contrast of the sequence is low, especially without the use of contrast-agents, which is why for segmentation angiogram-like images are generated from the absolute velocity. These PC-MRAs can be calculated in a time-resolved way, but do not have sufficient signal in regions and time frames with low velocities, which is why they cannot be used for accurate, fully automated segmentations (see Figure 3B). 3D segmentation is therefore done in a semi-automatic way for static images and there is a strong need for fast, robust and automatic delineations.

Classifying machine learning tasks like the U-Net (62) have been used broadly to define labels and their location in 2D or 3D images. They are built up by an encoder part, so the down-sampling of spatial information, and a decoder part, restoring the spatial information. The networks are trained on paired datasets of the original image and the matching voxel-based segmentation. To compare the geometric segmentation results the DS and the Hausdorff distance (HD) are used. The DS ranges from zero to one and calculates the voxel-based match between learned and ground truth geometry. The HD is the maximum value of all (Euclidian) distances calculated between each point of a geometry and the closest point of another geometry.

Bratt et al. (63) trained a U-Net to segment the aortic valve from 2D flow MRI magnitude images, based on manual, time-resolved segmentations. They achieved a DS of 0.94 using 150 aortic datasets for training. In 190 additional testing datasets (patients with coronary artery disease) the ML based segmentation demonstrated high correlations in the analysis of net forward flow through the aortic valve when compared to a manual delineation (*r* = 0.99, *p* < 0.001) and it performed better than a commercial automatic segmentation [significant differences in flow 1.85 ± 1.8 ml (U-Net) vs. 3.33 ± 3.18 ml (automatic)]. Also, in a different patient cohort with BAV and stenotic aortic valves acquired at a different scanner and vendor the network performed equally well in comparison to manual segmentations (correlation *r* = 0.99, *p* < 0.001). In a similar study, Garcia et al. (64) trained a network to detect and track the movement of the aortic and the mitral valve in 3-chamber cine (bSSFP) images. The resulting position of a 2D plane through the valve was interpolated onto 4D flow MRI data acquired in the same scan session in 106 subjects resulting in significant differences in flow and peak velocity between aortic- and mitral valve disease patients and controls (no comparison between manual and ML segmentation was conducted). Tsou et al. (65) trained two networks, a MultiResUNet (66) and a U-Net to perform 2D contour delineations of the cerebral aqueduct on 333 (266 training, 67 validation) cerebral 2D flow MRI datasets. Cerebrospinal fluid flow through the aqueduct was similar for both segmentation approaches when compared to segmentations of a radiologist. The DS was slightly higher for the MulitResUNet than for the U-Net (DS = 0.933 vs. DS = 0.928, respectively) and the MulitResUNet was less prone to segmentation errors than the U-Net.

In 2020, Berhane et al. (67) used 4D flow MRI scans of a wide range of age, body mass index and aortic valve types of 1,018 subjects (528 BAV and 376 tricuspid aortic valves, 114 healthy controls) to train a CNN based 3D U-Net (62) segmentation network for labeling the aorta in a systolic timeframe. Training datasets were constituted from manually labeled images, done by >20 operators. The segmentations resulted in a DS of 0.951 and HD of 2.8 mm for the testing dataset (499 training, 101 validation, 418 testing). Additionally, a centerline was automatically detected, and perpendicular slices were chosen with the vessel boundary being the segmentation. These values were then compared against each other in peak velocity (< 0.001 m/s, LOA 0.01% for the CNN at all regions) and net flow (–0.2 to 0.1 mL/cycle, LOAs 6.4–9.2%) to quantify differences. Interestingly, most deviations in the testing cohort with a DS below 0.9 were around the aortic outflow tract or at the superior extend of the aortic branches, indicating a difference in the segmentations extend. Also, the CNN achieved DS similar to the interobserver values (DS = 0.95). This ML workflow can certainly be used on a wide range of 4D flow MRI images, eventually it requires retraining if different PC-MRA calculation methods are used. Similarly, Garrido-Oliver et al. (68) trained a 3D nnU-Net (69) for static segmentations of the aorta and a Deep Q-Network (DQN) (70), based on reinforcement learning, for landmark detection on 323 patients (BAV, genetic syndrome, aneurisms) who received 4D flow MRI scans. For the aortic segmentations they achieved a DS of 0.949. The landmark detection algorithm performed well in the identification of the supra-aortic vessels, and it performed less good in the detection of the sinotubular junction and the pulmonary artery bifurcation. The sinotubular junction, however, was also challenging to be identified by human observers. Both studies, (67) and (68), did not take the motion of the aorta into account but included only time-averaged images.

So far, only limited studies exist on training time-resolved segmentations from 4D flow MRI. However, time-resolved segmentations are of interest when investigating stiffness by PWV (71–74), and to avoid inaccuracies in flow estimation, as the aortic root can move up to 8 mm within one heartbeat (75). Segmentation of time-resolved images is very time-consuming as it requires a 3D segmentation for each of 10–40 timeframes. In 2022, Bustamante et al. (76) created a framework to segment all 4 cardiac chambers, the aorta, and pulmonary arteries for all time frames from contrast-enhanced 4D flow MRI data. A 3D U-net was developed on 205 4D flow dataset (144 training, 20 validation, 41 testing), which contained a variety of cardiac disorders (*N* = 165). Forty cardiac frames were acquired and treated as independent segmentations. The segmentations were compared against ground truth, manually corrected, atlas-based segmentations also developed by Bustamante et al. (77). This method registers a general segmentation mask onto the image, which is, however, computationally expensive. The results showed good overall scores, the best scores achieved in the aorta. Time-averaged DS were >0.9 for all anatomies, similar to Berhane et al. (67).

To avoid the problem of poor myocardium-to-blood contrast in 4D flow MRI and time intensive pre-registration on atlases, Corrado et al. (78) used a stack of 2D time-resolved short-axis cine (bSSFP) images acquired at the same scan session to segment 4D flow MRI of mainly healthy subjects (*N* = 105). They used a pretrained fully convolutional network (FCN) from Bai et al. (79) trained on 4,875 short axis bSSFP images of the UK biobank study to create a 3D segmentation of the left and right ventricle. Then a 3D-to-3D registration of the time-averaged bSSFP and 4D flow data was done to map the segmentation results onto the 4D flow dataset. The automated segmentation (LV: DS = 0.92, RV: DS = 0.86) showed good agreement with manual segmentations (LV: DS = 0.91, RV: DS = 0.87).

Corrado et al. (80) also developed a ML based plane selection (80), which automatically defines measurement planes perpendicular to the 8 great vessels: ascending aorta, main pulmonary artery, superior and inferior vena cava, and the 4 pulmonary veins. The training was done on 323 subjects (241 training, 42 validation, 40 testing; in total 186 healthy controls, 123 patients and 14 with unknown health status). A 3D CNN predicted the probability of a predefined patch (32 × 32 × 32 voxels) containing a vessel and also location, size and a double oblique plane on that vessel. The CNN was based on residual learning [ResNet (81)] with residual blocks for feature extraction and convolutional blocks for downsampling. At each plane either done by ML or manual selection, a segmentation of the vessel was performed automatically based on the PC-MRA and net flow was calculated and compared. As a result, the correlation between the ML algorithm and two manual observers was slightly lower (observer 1 vs. algorithm: *r* = 0.68 and observer 2 vs. algorithm: *r* = 0.72) that the difference between the two observers (*r* = 0.81). Also, the algorithm was more accurate on straighter vessels such as the SVC and worse in the ascending aorta. The performance was stable for all flow estimations (as this was probably insensitive to small variation in measurement plane). Also, the patient datasets were an additional challenge for the network suggesting more diverse datasets. Overall, the ML method was faster than atlas-based approaches. Processing times when applying the ML were 18s vs. 300–400 s for a manual observer. The study suggested a reinforcement learning approach for measurement plane planning in the future.

Contrast enhanced 4D flow MRI is used for many clinical examinations and creates a better blood-tissue contrast than conventional 4D flow MRI. In medical imaging, realistic but fictitious images can be produced by generative adversarial networks (GANs), and CycleGANs (82, 83) in particular. Bustamante et al. (84) used a cyclic GAN, to artificially transform non-contrast cardiac enhanced scans into contrast enhanced data. The cyclic GAN can be considered as unsupervised learning which needs two images sets as input, which do not have to be exact pairs. It consists of two generators or data transformation functions that transform (1) non-contrast data into contrast data and (2) contrast data into non-contrast data. It also consists of two discriminators that distinguishes (1) artificial from real contrast data and (2) artificial from real non-contrast data. They used 69 with and 72 datasets without contrast agents for training a 2D GAN. In total additional 81 non-contrast aortic datasets were used for testing and were converted into artificially enhanced datasets using the GAN. For training, the data was cropped and rearranged as 120 2D slices in a coronal view, using only the magnitude image as an input. The quantitative evaluation of the artificially enhanced test data showed an increase in contrast-to-noise ratio (CNR) by 88%, and an increase in SNR by 48%. This was achieved while maintaining a structural similarity index, describing structural information, of 0.82 ± 0.01 and a mean relative error of 0.09 ± 0.01 between enhanced and original images. Also, segmentation on artificially enhanced data performed better than on regular data.

## Statistical evaluation of blood flow

ML has the potential to support the statistical classification of healthy controls and patients with cardiovascular disease based on 4D flow MRI data using supervised or unsupervised learning. For classification, typically a set of hemodynamic features is derived from the data (such as velocity, vorticity, etc.), then the number of features is reduced by a feature-selection step e.g., using a sequential forward search. A set of different classifiers is then tested during (supervised/unsupervised) training and the best performing features, feature-selection steps and classifiers might be used for future predictions.

Niemann et al. (85) developed a method for feature-based classification of patients with BAV and healthy controls, based on aortic 4D flow MRI. They trained a network to classify between (1) BAV (*N* = 22) and healthy controls (*N* = 90), (2) BAV and “older” healthy controls (*N* = 30) and (3) male and female subjects. Their framework included hemodynamic feature selection, model training and hyperparameter tuning. Selected features were parameters such as minimum, maximum and mean velocities derived from planes perpendicular to the aortic centerline. Classifiers used for training were methods such as random forest (RF) and support vector machine (SVM). The results for classifying the task were for (1) an accuracy of 93% with features time-to-peak vorticity, time-to-peak in-plane velocity and peak-systolic in-plane mean velocity using sequential forward search (SFS) as a feature-selection method and RF as a classifier, for (2) an accuracy of 100% with features peak-systolic mean velocity, time-to-peak-systolic-through-plane mean velocity and diastolic median right rotation volume using SFS as a feature selection method and SVM a classifier, and for (3) an accuracy of 69% with features peak velocity, peak systolic velocity and time-to-peak-systolic-through-plane velocity using SFS as a feature selection method and RF as a classifier. The results of the classification model demonstrated a good distinction between BAV and controls and only moderate distinction between male and female subjects. Also, in Franco et al. (86) the hemodynamics of the thoracic aorta in 4D flow MRI data of patients with BAV was analyzed searching for new biomarkers. The aim was to find a ML model that distinguishes three classes: BAV patients with (*N* = 49) and without (*N* = 18) dilated ascending aorta and healthy controls (*N* = 48). A total of 17 hemodynamic features such as e.g., forward velocity, velocity angle, vorticity, KE, TKE and WSS were extracted from 4D flow MRI data in two parts of the aorta. Then a set of classifiers (linear discriminant analysis, k-nearest neighbors, quadratic discriminant, Mahalanobis distant, SVM, neural network, RF) were tested and used to train a neural network with multiple layers. The performance was evaluated with repeated cross-validation and Pearson correlation between the hemodynamic features. Overall, the model classifying the data showed, that linear discriminant analysis (96.3% accuracy) and random forest (96.0% accuracy) were the best performing classifiers using the features: velocity angle, forward velocity, vorticity, and backward velocity in the ascending aorta.

## Conclusion

Current 4D flow MRI acquisitions are constrained by their scan time, spatio-temporal resolution, and SNR, limiting their accuracy and clinical application. Semi-automatic post-processing steps, including phase corrections and segmentation for vessel delineation are time-consuming and in need for automation. This review shows various ways of accelerating image reconstruction times and post-processing tasks using ML, when compared to the current state-of-the-art approaches. Code and data have been made publicly available for many ML applications reviewed for this article (as summarized in Table 1), which supports their reproducibility, applicability and development. A table summarizing all papers reviewed and their technical details can be found in the Supplementary Table 1.

**TABLE 1:** Available code for all original research papers screened for this review.

References	Topic	Code
Vishnevsky et al. (28)	Reconstruction of undersampled Cartesian 4D flow MRI data (aorta)	https://codeocean.com/capsule/0115983/tree
Haji-Valizadeh et al. (39)	Reconstruction of radial 2D flow MRI data (aorta)	https://dataverse.harvard.edu/dataset.xhtml?persistentId=doi:10.7910/DVN/N97M6H
Kim et al. (40)	Fast 4D flow MRI by estimating velocity maps from 3-point encoding	https://github.com/uwmri/ThreePoint4DFlow
Ferdian et al. (41)	4D flow MRI super-resolution framework	https://github.com/EdwardFerdian/4DFlowNet
Kissas et al. (49)	1D flow physics informed DNN	https://github.com/PredictiveIntelligenceLab/1DBloodFlowPINNs
Ferdian et al. (54)	WSS estimation from 4D flow MRI	https://github.com/EdwardFerdian/WSSNet
Berhane et al. (61)	Anti-aliasing correction of 4D flow MRI data	https://github.com/hberhane/4D-flow-Velocity-Aliasing-CNN
Bratt et al. (63)	Segmentation on 2D flow MRI data	https://github.com/akbratt/PC_AutoFlow
Tsou et al. (65)	Segmentation on 4D flow MRI data	Uses MultiResUNet from (66): https://github.com/nibtehaz/MultiResUNet
Corrado et al. (80)	Automatic measurement plane selection on 4D flow MRI data	https://github.com/pcorrado/DL-Vessel-Localization
Corrado et al. (78)	Ventricular segmentation on 4D flow MRI data	Using the FCN from (79): https://github.com/baiwenjia/ukbb_cardiac
Garrido-Oliver et al. (68)	3D segmentation and landmark detection 4D flow MRI data (aorta)	Uses the nnU-Net (69): https://github.com/MIC-DKFZ/nnUNet Reinforcement learning and landmark detection: https://github.com/CardiovascularImagingVallHebron/4D_flow_landmark_detection

In the future, it will be essential that accurate cardiovascular 4D flow MRI can be performed in a single, fast scan. That includes an easy choice of VENCs (by retrospective correction of anti-aliasing and phase offsets) and spatio-temporal resolutions that might be increased by super-resolution approaches retrospectively to the scan and for vessels with slow flow and small geometries. It is important, that the analysis of the data is performed in an automated, operator independent and robust way, to allow accurate assessment of biomarkers such as peak velocities and WSS for diagnosis and clinical decision making. Classification of disease by 4D flow MRI-derived biomarkers has the potential to be reinforced by ML technologies.

## Author contributions

EP conceptualizing of the manuscript, literature research for manuscripts included in the review, and manuscript writing. PO, BJ, and JB conceptualizing of the manuscript, technical feedback and discussion, and manuscript reviewing. AH and CG clinical feedback and discussion and manuscript reviewing. All authors contributed to the article and approved the submitted version.

## Supplementary material

Supplementary Table 1 All papers reviewed and their technical details.
