# Supplementary figures and images for: Advances in machine learning applications for cardiovascular 4D flow MRI
Source: Front Cardiovasc Med. 2022 Dec 9;9:1052068. doi: 10.3389/fcvm.2022.1052068 (PMC9780299; doi:10.3389/fcvm.2022.1052068)

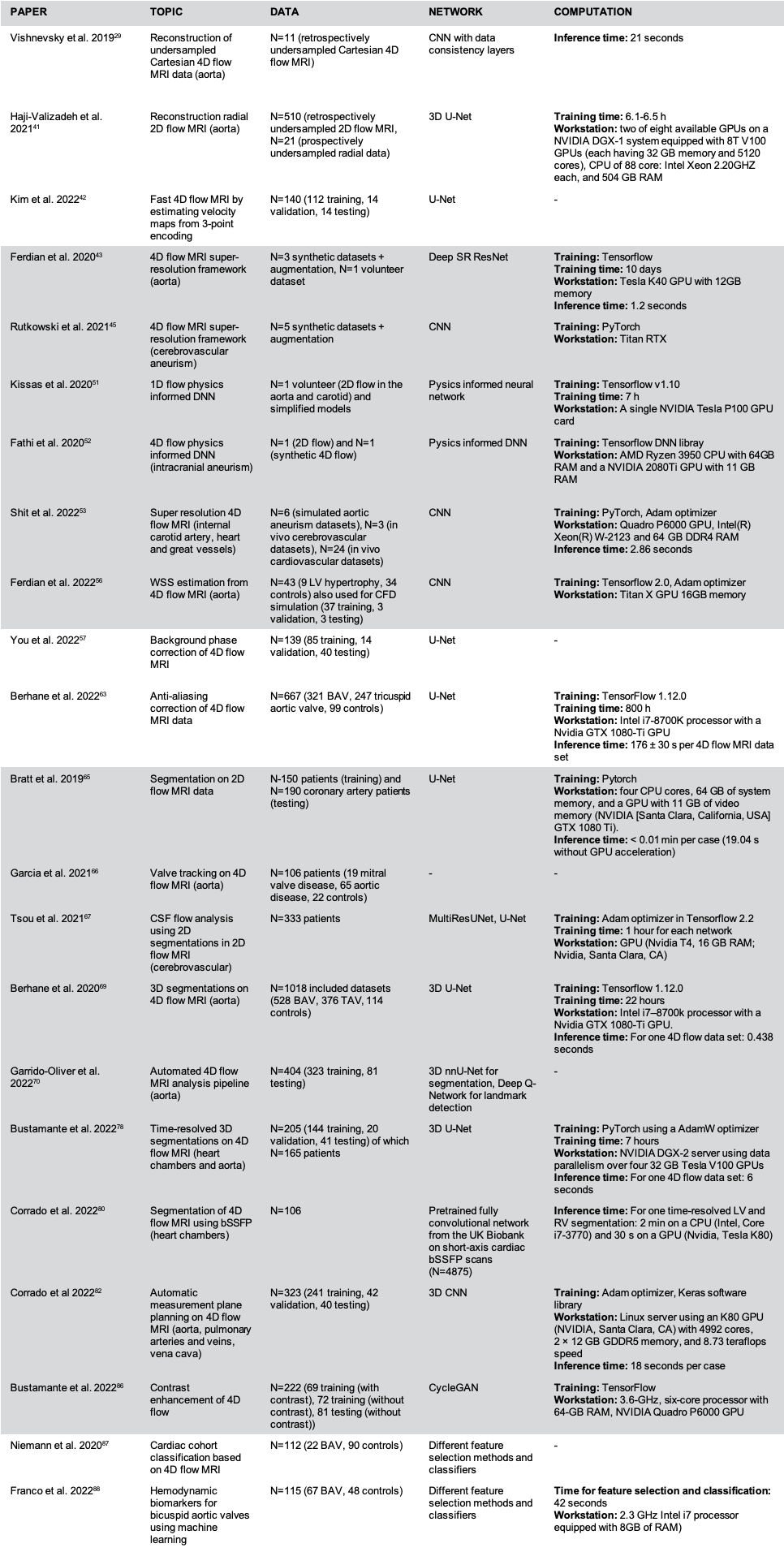

Supplement: Supplementary Table 1 — All papers reviewed and their technical details. [file Image_1.PNG]
